# Women’s experiences of a randomised controlled trial of a specialist psychological advocacy intervention following domestic violence: A nested qualitative study

**DOI:** 10.1371/journal.pone.0193077

**Published:** 2018-11-27

**Authors:** Maggie Evans, Alice Malpass, Roxane Agnew-Davies, Gene Feder

**Affiliations:** 1 Centre for Academic Primary Health Care, Bristol Medical School, University of Bristol, Bristol, United Kingdom; 2 Domestic Violence Training Ltd., London, United Kingdom; Stellenbosch University, SOUTH AFRICA

## Abstract

**Background:**

Women’s experience of domestic violence and abuse (DVA) is associated with mental illness which may not be addressed by domestic violence advocacy. The study aimed to compare the experiences of women receiving a psychological intervention with women receiving usual advocacy in a randomized controlled trial (PATH: Psychological Advocacy Towards Healing), to illuminate the trial results by exploring women’s experiences of benefits and difficulties.

**Methods:**

A qualitative study nested within the PATH trial, based in two DVA agencies in the UK. A purposive sample of thirty-one intervention and usual care participants were interviewed up to three interviews over a year. Thematic analysis was carried out, incorporating concepts from the Trans-Theoretical Model of change.

**Findings:**

The PATH trial reports a clinically relevant improvement in mental health outcomes for women receiving the intervention compared to usual advocacy. The qualitative study reveals which elements of the intervention were beneficial or problematic, which outcomes were most meaningful and relevant to participants and highlights reasons for variations in adherence. Women valued the educational, psychological and emotional elements of the intervention, they felt safe to explore repressed emotions for the first time and experienced a reduction in self-blame, improved sense of identity and greater self-esteem. They also incorporated new skills and self-help techniques to enable sustainable change. Women receiving usual advocacy reported un-met needs for psychological and emotional support. Adherence was affected by women’s ‘psychological ‘readiness’ to engage, the competing demands of practical issues such as housing insecurity, legal proceedings or the availability of child care, and breaks in the continuity of professional care.

**Conclusions:**

Continuity and regularity of sessions with a trained specialist worker was key to women’s recovery. Individual assessment of ‘readiness’ would optimise the timing of delivery to maximise adherence and benefit.

## Introduction

Domestic violence and abuse (DVA) can be physical, sexual, psychological and economic, perpetrated by a partner, ex-partner or adult family member. Women experiencing DVA have increased risk of depression, post-traumatic stress disorder, substance use and suicidality [1,2,3] but their mental health needs are not generally met by standard psychological interventions such as counselling and cognitive behaviour therapy [4]. Treatments that do not directly address the violence may be ineffective or even harmful [5,6,7]. In-depth studies of recovery from DVA reveal the importance of promoting self-empowerment and resilience amongst women, to protect against future abuse [8–10].

In the United Kingdom and the United States, many women who experience DVA self-refer or are referred to specialist agencies where advocates provide practical and emotional support, including safety planning and access to crisis accommodation. Qualitative studies of women experiencing DVA highlight the importance of psychological as well as practical support to promote sustainable change and to help women stay free of abuse [11–13]. However, DVA advocates are not usually trained to address mental health needs.

The Psychological Advocacy Towards Healing (PATH) trial, published alongside this article, aimed to improve psychological functioning and mental health for women experiencing DVA through a specialist psychological intervention delivered by specially trained DVA advocates [14]. Details of the intervention are provided in the accompanying article and our protocol paper [14, 15]. Here we report the findings of a qualitative study nested in the trial.

Qualitative studies in trials have an important role in interpretation of trial findings and increasing our understanding of how contextual barriers and facilitators may influence outcomes, [16–21]. Understanding context is critical in interpreting the results of a trial and generalising beyond it, enhancing external validity [17, 20, 21]. Insights from qualitative research can also inform implementation if the intervention is successful and can help trialists ‘to be sensitive to the human beings who participate in trials’ [20]. Another value of qualitative work is the application of social science theory in understanding mechanisms of change [17]. The design of the nested qualitative study and the interpretation of data were informed by a modified version of the Trans-Theoretical Model of Health Behaviour Change [22]. In this qualitative study our primary aim was to identify outcomes most valued by participants and to explore barriers and facilitators to adherence to the intervention. A secondary aim was to compare the experiences of women receiving the intervention alongside usual DVA advocacy with those receiving advocacy alone.

## Methods

We recruited a purposive sub-sample of thirty-one PATH trial participants across a range of age, income and ethnicity, with and without children, based in safe houses (shelters) and in the community, and from both arms of the trial, which was based in DVA agencies in two cities in the United Kingdom. The PATH intervention was designed by RAD. DVA advocates from two participating agencies were trained by RAD over a period of three months to deliver up to ten one-to-one weekly sessions with women seeking support for DVA. The content was psycho-educational and included help to recognise abuse, an opportunity to explore personal experiences and on-going affects of abuse and to learn self-help strategies.

Recruitment took place between May 2011 and March 2012. Participants were approached face-to-face and, following written informed consent, they were invited to take part in a maximum of three semi-structured interviews: at the beginning and end of the intervention and after one year of follow-up, with equivalent times for usual care participant The number and spacing of interviews aimed to be responsive to the availability and the experiences of the women, which varied for each individual. We aimed to collect sufficient data to fulfil the study aims, reaching data saturation where possible.

An advisory group of women survivors of DVA, gave advice on the development of information leaflets, topic guides and study processes, and provided input into the interpretation of data.

ME an experienced female researcher, conducted the interviews, following a topic guide (Table 1: Topic Guide(Qualitative interviews). Interviews took place at a safe location where the risk of contact with the perpetrator was minimised. Locations included a local authority housing office, a healthcare centre, the home of a participant’s relative or the participant’s own home. Interviews were audio-recorded and transcribed verbatim.

**Table 1:** Topic guide (Qualitative Interviews).

Topic	Questions
Background	Details of living situation, children, contact with family and friends, employment and financial situation
	Brief history of abusive relationships, current location of perpetrator, personal safety
Help-seeking	Triggers / decision-making about contacting agency / other avenues tried
	Expectations of agency and help obtained
	Disclosure / help-seeking with family / friends
General Health History	Physical, emotional, mental health
	Use of health services
PATH or advocacy sessions	Timing and relevance
	Expectations
	Benefits / problems
Relationship with SPA or support worker	Trust and Safety
	Quality of relationship
	Likes / Dislikes about person / process
Attitude towards change	Turning points
	Main sources of help
PATH or advocacy sessions: strategies / homework	Elements of intervention most and least valued
	Relevance of specific techniques / hand-outs
Overview	Self-esteem
	Ability to make choices
	Main benefits / problems
	Any issues with adherence
Experience of taking part in a research study	Knowledge and understanding of research
	Motivation to take part
	Positive / Negative experiences

The data were coded and thematic analysis was carried out using the qualitative software NVivo. The coding strategy and themes were developed using an iterative process involving all the authors. Reading, re-reading and coding of transcripts was carried out by ME and KB in close collaboration. Attention was given to the exploration of contradictory and minority themes. Data from repeat interviews were used to develop narratives for individual cases [23]. Verification of the emerging codes was provided by double coding of some transcripts by ME and KB. GF and RAD also part-coded some transcripts. Themes were identified from the study questions and others emerged from the data. The constant comparison approach was used to continuously refine the themes and re-code earlier transcripts. Consensus was gained by all authors and the Advisory Panel who provided verification and credibility checking of the themes and the data interpretation. All authors were unaware of the trial results during the qualitative data analysis to avoid bias in data interpretation [17,18]. The findings are presented here with knowledge of the quantitative results, to enhance our interpretation of trial outcomes.

The study was approved by the UK South West National Research Ethics Service. Trial Registration: ISRCTN58561170. The application for trial registration was submitted to ISRCTN on 23.02.2011. Registration was delayed because ISRCTN suggested a fee waiver for National Institute for Health Research funded studies that are adopted into the NIHR portfolio. The adoption process was unexpectedly lengthy and delayed the finalisation of ISRCTN registration, confirmed on 26.07.2011.

## Results

### Characteristics of the sample

We recruited twenty-one women from the trial intervention group and ten from the usual care group (Table 2). A further four women who consented to contact could not be traced. Twelve of the intervention group completed the full course of ten SPA sessions (‘completers’). Nine women attended between one and seven sessions, with an average of 4.5 [‘non-completers’]. The number and spacing of qualitative research interviews varied according to the availability of participants (Table 3).

**Table 2:** PATH qualitative study participants (N = 31).

Qualification	N	Employment status	N	Age	Children	N	Ethnicity	N	Length of time in most recent abusive relationship
School	12	Employed	8	Mean age 35 yrs	Yes	26	White British	25	Mean 8 yrs
University	5	Looking after children	8	Range 20–65 yrs	None	5	Iraqi	1	Range 1.5–30 yrs
Other	10	Unemployed	8				Spanish	1	
None	4	Long-term sick	4				Black Caribbean or Mixed race	4	
		Retired	2						
		Full-time education	1						

**Table 3:** Number of interviews carried out.

	Interviewed once	Interviewed twice	Interviewed three times	Reasons for <3 interviews
**SPA completers**	0	4	8	Lost to follow-up (2) Declined third interview (1) Data saturation reached (1)
**SPA non-completers**	4	5	0	Lost to follow-up (4) Declined third interview (2) Data saturation reached (3)
**Usual care**	4	4	2	Lost to follow-up (4) Unwell (1) Data saturation reached (3)

This study was nested within the PATH trial (Psychological Advocacy Towards Healing) that reports a clinically relevant improvement (article submitted). This qualitative study illuminates these results by revealing elements of the intervention women regarded as either beneficial or problematic and by giving insights into outcomes that were most meaningful and relevant to them. Their accounts reveal the complexity of the therapeutic processes and the range of benefits experienced by participants.

The results are presented in two sections. First, the experience of intervention and usual care participants are compared. Second, the experiences of ‘completers’ and ‘non-completers’ of the intervention are compared, to gain insight into barriers and facilitators to adherence to the intervention, and to further our interpretation of the Complier-average Causal Effect (CACE) analysis in the trial. This showed greater benefits for women who attended more sessions, proportional to the number attended.

The findings are illustrated by verbatim quotations from participants **(see Supporting Informaiton** S1 Text. **Verbatim quotations from participants)**.

### Comparison between intervention and usual care groups

We identified specific elements of the intervention and specific outcomes which women considered the most important and meaningful. This provides a more nuanced understanding of the potential benefit of the intervention over usual (advocacy) care, including benefits that were not captured by trial outcome measures.

#### (i) Relationship with SPA or advocate

The most important aspect of the intervention for participants was their relationship with their SPA or advocate, who had knowledge and understanding of DVA gained through their work and, sometimes, from personal experience. Women welcomed the chance to talk to someone, outside their network of friends and family, who was non-judgmental, and was offering help. Following the initial relief of making contact, however, the experience of women in the two groups diverged over time. Women receiving the intervention valued the regular, predictable pattern of SPA sessions once a week or a fortnight, with flexibility around child-care and school holidays, alternating with advocacy sessions. Women receiving usual care were dissatisfied with the less regular, less predictable contact they experienced.

In the intervention group, women described a close, confiding relationship with their SPA, which was a crucial part of their PATH experience. SPAs were described as compassionate, warm, empathic, calm, intelligent and thoughtful; like a trustworthy friend with whom the women easily bonded, with no sense of the professional distance they had anticipated from their image of a ‘counsellor’.

The data reveal that SPAs modelled a caring, person-centred, affirmative and non-judgmental relationship, contrasting sharply with women’s everyday experience of an abusive relationship. Rapport and trust were built through shared laughter and banter. Women valued their SPA’s astuteness in identifying and challenging, rather than colluding with, their automatic, ingrained patterns of behaviour, such as self-deprecation or the use of tactics to avoid facing the reality of abuse. Women were encouraged to get to the heart of issues, to identify and release painful emotions. SPAs suggested strategies to avoid abuse in the future.

A rare contrasting case was provided by one PATH ‘completer’ who felt her SPA was reserved, with limited ability to provide emotional support or to help her explore her feelings in depth.

In the usual care group, omen became aware, over time, of the limited availability of their advocate, and their lack of control over the timing or frequency of her visits. Women said their advocates primarily gave practical support for re-housing, financial, legal or child-care issues, rather than psychological and emotional support, and tended to give quick unhelpful responses to their distress. In contrast, women receiving the intervention talked about the time, attention and emotional exploration offered by their SPA. At follow-up, very few usual care women felt they had received emotional as well as practical support from their advocate, and many felt let down by only having ‘occasional chats’ rather than more substantial psychological support or counselling.

#### (ii) Emotional and cognitive outcomes

All participants described anxious or depressed feelings at first interview. The majority had been prescribed anti-depressants but few had taken them. Some women had previously experienced counselling without a specific focus on DVA, which had limited usefulness, and some felt stigmatised when offered counselling via mental health services.

At follow-up, few women in the intervention group spontaneously mentioned improvements in mood as a key outcome, although after prompting more women did report a reduction in mood swings, panic attacks, or anxious and depressed feelings. More important outcomes for them were the ability to identify and understand patterns of abusive behaviour, which led to an increase in confidence, self-esteem and sense of identity, and a reduction in self-blame. They reported feeling more assertive, better able to trust and communicate with others. Improved mood was perceived as contingent on these more fundamental changes. Cognitive changes were also valued, such as learning techniques to curb negative automatic thoughts, to develop a more positive outlook, and to recognise the warning signs of DVA in order to avoid future abuse.

Women also described feeling more comfortable talking about and caring for themselves rather than prioritising others, more able to express their emotions rather than repressing them or drinking alcohol. They also experienced improved concentration and better sleep. Significant markers of changes included improved communication and relationships with family members including their children and, for some, increased confidence to seek social contacts, employment or further support. Some women had difficulty persisting with new patterns of thinking and behaving in the face of on-going harassment, but were learning to respond differently, for example by not answering texts from the perpetrator.

Whether or not they completed all the sessions, women described integrating new understanding and skills learnt from their SPA into daily life at one-year follow-up. They continued to use hand-outs and techniques and recommended them to others.

In contrast, in the usual care group, women’s narratives were dominated by their ‘physical trajectory’, moving to a safe location, returning to work or to college, but without a sense of moving on emotionally or psychologically. Unable to identify or break deeply ingrained responses to abuse, women continued to self-blame, experienced difficulty asking for or accepting help, and felt unable to escape on-going harassment. Rarely, participants described learning useful self-help techniques from their advocate; for example dealing with stress by going for a walk, having a relaxing bath with candles and phoning someone for a chat.

At follow-up, with very few exceptions, usual care women highlighted the lack of emotional-psychological support as a key deficiency in service provision, and recognised their on-going need for some kind of counselling. Some were on long waiting lists.

#### (iii) Identifying key elements of the intervention

Choice and flexibility emerged as key elements valued by participants. Women varied in their preferences for particular techniques and topic areas, but valued being offered choice, which represented a stark contrast to an abusive relationship where choice is very limited. SPAs tailored sessions to individual needs and suggested hand-outs and exercises for continuity between sessions. Women valued many techniques including writing down feelings, saying affirmations, writing a letter to the perpetrator to later destroy, keeping a journal, talking to the perpetrator as if he was in the room, relaxation and visualisation, self-nurturing, and expressing anger using experiential techniques. A favoured technique was to write down difficult thoughts or feelings, put them into a real or imaginary box and store them until they were ready to look at them or destroy them in order to move on. Some women disliked some or all of the PATH techniques, particularly role play and anger work, and were encouraged to find their own self-help strategies such as spending time with a favourite pet, or taking long walks.

### Comparison between PATH ‘completers’ and ‘non-completers’: Factors affecting adherence

We conducted follow-up interviews with seven of the nine interviewees who did not complete all SPA sessions. ‘Non-completers’ spoke positively about their early experience of SPA sessions at first interview. At follow-up many said they had worked through important issues, gained a better understanding of DVA, learned coping strategies and were ready to move on without completing all the sessions. Other ‘non-completers’ felt the psychological process was unfinished and they hoped to continue at a later stage.

‘Non-completers’ gave three main reasons for not attending the complete course: ‘*emotional stress’*, *‘psychological desire to move on’* and ‘*lack of continuity with SPA’*. An additional theme of *‘level of engagement’* emerged from the data.

#### Emotional stress

Some women felt the intervention was too emotionally draining at that point in their lives. They described feeling overwhelmed after a SPA session and too vulnerable to deal with pressing concerns such as the demands of young children or a pending court case. This sense of vulnerability was exacerbated by a perceived lack of support in between sessions when SPAs were not easily available.

#### Psychological desire to move on

Some non-completers wanted to focus on the future and put the experience of DVA behind them. SPA sessions served as a reminder of past distress and bad memories, ‘digging up what I’ve buried'. Some women preferred to stick to old coping strategies such as blanking out painful feelings, or felt that recovery from abuse was a matter of time rather than intervention.

#### Lack of continuity of SPA

As a corollary to high investment in their relationship with their SPA, women felt easily let down if there was a break in continuity of contact. This was a major reason for failing to complete SPA sessions. Three vulnerable time points emerged. First was the gap between one SPA session and the next, when some women wished for easier access to their SPA by telephone or in person. Second was any break in the continuity of sessions or a change of SPA owing to maternity leave, illness or a SPA leaving the service. Third was loss of contact if a participant moved house or lost her mobile phone.

Nine of the twenty-one intervention women experienced a break in continuity, without adequate explanation. This left them feeling vulnerable and uncertain as to what was happening next. If contact with the agency was eventually restored, some women went on to complete the full course of sessions. Others were unwilling to re-engage, either because it meant starting with a different SPA or they felt they had already ‘moved on’ in the intervening time.

When the full course of sessions ended, ‘completers’ generally felt well prepared for its ending and inevitable separation from their SPA, marked by an exchange of gifts. A minority would have preferred a more gradual weaning from their SPA over a longer time and one ‘completer’ felt that she had lost her ‘link to life’.

#### Levels of engagement

Women varied in their preference, from ‘deep’ emotional engagement with SPA sessions to a preference for learning everyday skills and strategies. Our data suggest that women who engaged more deeply were more likely to complete their sessions. Completers were also more likely to have been longer in an abusive relationship (mean: 11 years) than ‘non-completers’ (mean: 4.5 years), and were more likely to have experienced multiple abusive relationships, in childhood, adulthood or both [see Table 3]. In contrast, women without historic abuse were generally less keen on ‘deep’ emotional work and preferred to engage at the level of learning new skills and self-help strategies. They did not always feel the need to complete the full course.

Contextual factors limited adherence to the intervention for some women, for example the constraints of child-care, the emotional and practical demands of parenting and working, or the busy, chaotic environment of a refuge with little personal space. Women at home owing to poor health or retirement, without young children, had fewer competing demands and greater availability to fully engage with the intervention.

Women completing the full intervention course found it intense, rewarding and emotionally challenging; for some it was ‘life-changing’. They were, for the first time, able to identify, acknowledge and express long-repressed anger, grief and fear, and to disclose recent and historic abuse. For some the relief was immediate; for others the benefits were gradual. The transformative power of the PATH intervention led many women on a powerful healing journey, conceptualised as weaving a silk tapestry, fitting missing pieces of a jig-saw, climbing up steps on a ladder or pushing a little bird out of the nest.

## Discussion

This qualitative study adds important detail to the PATH trial finding of a clinically relevant improvement in mental health outcomes for women receiving an intervention delivered by specialist psychological advocates (SPAs) compared to usual advocacy. The qualitative study reveals which elements of the intervention women regarded as beneficial or problematic and which outcomes were most meaningful and relevant to them, such as a reduction in self-blame, improved sense of identity and greater self-esteem. It also provides important insights into factors affecting adherence to the intervention. The findings will help to refine and target the intervention in future. Women’s accounts reveal the complexity of the therapeutic processes that resulted in wide-ranging benefits beyond or even in the absence of improvement in mental health status per se.

Women described benefits such as better understanding of DVA, increased self-confidence and improved coping strategies, which led to a reduction in mental health symptoms. Women receiving usual advocacy reported an un-met need for psychological support.

Women’s narratives suggest that an intervention combining educational, behavioural, emotional and psychological elements helped them deal with the emotional and psychological sequelae of DVA, with the potential for sustainable change. Participants highlighted the importance of delivery by DVA specialists rather than mental health specialists, which corroborates previous findings that referral for psychological counselling without a DVA focus does not necessarily meet the needs of these women [8].

Strengths of the study include repeat interviews over one year provided insights into short and long-term outcomes. The purposive sample enabled comparisons between intervention and usual care participants, participants with different levels of adherence, and the identification of contradictory views. The data were analysed without knowledge of the trial outcomes. Limitations include attrition meant that we could not trace ten participants for a final interview. There were only a few participants from minority ethnic groups.

Our qualitative analysis adds to the PATH trial findings, by identifying changes in psychological benefits that may be difficult to quantify, such as the provision of a safe place to explore previously un-expressed emotions, an improved understanding of DVA and the development of a more positive sense of self. Women learnt new coping strategies and felt more in control of their life and future. Comparing data from intervention and usual care participants suggests that PATH gives added value in relation to educational, cognitive, psychological and emotional outcomes compared to usual advocacy.

The trial reported improved outcomes for women with greater PATH adherence [14]. Insights into reasons for poor adherence are therefore crucial. The qualitative study highlights level of emotional distress, competing contextual issues, the psychological desire to move on and lack of continuity with the SPA as important factors. Receptivity to PATH may depend on where a woman is situated with regards to safety and freedom from fear, her level of distress and duration of abuse as well as her need to sort out immediate issues such as housing, legal proceedings and access to children. Our findings suggest that, for maximum benefit, attention must be paid to the timing of the intervention, following an assessment of individual ‘psychological readiness’ [13].

The concept of women’s ‘psycho-social readiness’ for change emerged from the application of the trans-theoretical model of change to DVA research. It highlights both the inner resources and external factors that need to be in place for a DVA survivor to engage with and benefit from an intervention [11,13,24,25,26]. Our qualitative data support the usefulness of the concept in explaining why some women engaged with and benefitted from the intervention more than others. Implementation of the PATH intervention could be targeted, following individual assessment or triage, to those most likely to benefit and at the most suitable time.

Although completion of a course of SPA sessions was important for maximum benefit, many ‘non-completers’ achieved important outcomes after attending less than ten sessions and did not wish to continue. Participants also expressed variation in their preferred level, or depth, of engagement. The potential to tailor the number and content of sessions to individual need could form part of the initial PATH assessment process.

Hearing the voices of DVA survivors is essential in designing and implementing interventions and services. The PATH model is a complex intervention comprising educational, behavioural, emotional and psychological elements. This study was carried out in the context of a large trial, which enabled access to a wide range of participants in two major cities in the UK. The results will be transferable to other urban contexts worldwide. The most highly valued feature for women receiving PATH was the working alliance with their SPA, a DVA specialist, embedded in an agency providing support and advocacy for DVA. Continuity of provision from a single advocate should be the gold standard, and any unavoidable transitions should be sensitively handled. Further research could explore the feasibility of adapting the PATH model for use in the community in order to reach the many women who do not access specialist services. Referral to a service based on the PATH model could form part of a package of options available via primary or secondary health care, based on the successful and widely implemented IRIS model [27].

This study in combination with the quantitative results, fidelity analysis and feedback from DVA professionals involved in the trial, will inform the future development and implementation of the PATH model.

## Supporting information

S1 Text Verbatim quotations from participants. (DOCX)
