# Supplementary material for: Women’s experiences of a randomised controlled trial of a specialist psychological advocacy intervention following domestic violence: A nested qualitative study
Source: PLoS One. 2018 Nov 27;13(11):e0193077. doi: 10.1371/journal.pone.0193077 (PMC6258524; doi:10.1371/journal.pone.0193077)
Supplement: S1 Text — (DOCX) [file pone.0193077.s001.docx]

**S1 Text. Verbatim quotations from participants**

**Relationship with SPA**

‘A (SPA) is brilliant, she’s absolutely brilliant , I can talk about stuff and it’s okay to talk about stuff. I used to feel incredibly guilty that I brought all this stress and trauma onto my children, my parents, my friends, my family…. I used to feel so guilty … you feel like you’ve done something wrong, that’s why he hurt me because I did something really bad, so therefore I must be a bad person in some way. The realisation of actually you’re not bad, it wasn’t your fault it happened. It’s like to just come to that point … we’ve had quite a lot of intense sessions and stuff, um, but to get to that point where you feel good about yourself is just amazing.’ **[SPA completer ID: 3015]**

‘P: I mean it’s sort of hard talking about like the rape and stuff… But that’s just generally hard to talk about anyway so… But A [SPA] was, was fantastic.

INT: Do you feel there are any bits of yourself or things that you held back on or do you feel you talked about everything really?

P: We talked about everything yeah.’ **[SPA completer ID:2064]**

**Relationship with advocate**

‘P: It’s been horrid. I don’t think, I’m still not through it and I don’t think I’ve had enough support. I don’t mean that in a horrible way to [DVA agency] because they have been absolutely fantastic but there’s something missing. Women in this situation being totally honest, need really strong counselling, really somebody there that they can turn to when they, do you know what I mean, even to just pick up the phone and just say is there any chance we can have a meeting in the next few days. Do you know what I mean? ….

INT: You couldn’t use your support worker in that way?

P: No, no because they are so busy. They’ve got so many people, there is so much running around and that to do.’ **[Usual care group ID:P2081]**

**PATH: Emotional and cognitive outcomes**

‘The good thing is that the questions that [SPA] asks me um and the sheet of questions that she’s given me as well, so I’m looking at them and answering when this happened, when that happened, how many times did he do this, did he say that, did he and so many things happen at the time of an attack that you forget an awful lot, well I think you choose to emotionally shut a lot of it out. .But this has opened up so many things um and helped me, those questions are so good because they’ve helped me to talk about a time that he did this or he said that and I’m thinking oh my god he actually did use to do that quite often really, not rarely, it happened quite a lot and I think most days and I’d think my god and I didn’t even realise at the time. So the questions have helped hugely because they’ve helped me become more accepting of the fact that it was abuse, the control was abuse.’**[SPA Completer ID: 3015]**

‘I think they’re one of the steps of the ladder, they were so important and they were relevant to where I was then, at that stage … but it seems that I’m moving on in the journey…. I’m surprising myself on a daily or weekly basis that I’m just, em, feeling bright and chirpy about things, but I’m also very aware that I’ve got, I’m struggling with issues still … Yeah, she really did help me, she really gave me that little, you know, it’s like pushing a little bird out of the nest and just … I think it was about right [timing], I think, em, I would have perhaps liked it a bit longer, but that would have been my neediness, em, my, em, insecurity.’**[SPA completer ID: 3044]**

**PATH: Ending sessions**

‘She said to me, um, we’re going to say goodbye to each other, when we got to a certain stage and that, and she said think about something that you’d like to bring to me and I’m going to bring something to you, so it’s like a goodbye thing. So, um, I brought here, I brought a big bar of chocolate and some Red Bull to [SPA]. She said to me, ‘Okay,’ so I said, ‘well, the chocolate is for the feel good and the Red Bull is because you’ve given me back energy, I’ve got myself back.’ **[SPA completer ID: 3015]**

‘If you’re in refuge at the time ... it gets stressful when you ... you ... you leave and you go into your own accommodation and that ... I’d seen [SPA] for the last time and then all of a sudden I had all this stress with moving and ...I didn’t see her for months, and it would have been nice to have her ... there, you know.’ **[SPA completer ID: 4003]**
